# Tyrosine kinase inhibitors in HER2‐positive metastatic breast cancer with trastuzumab emtansine resistance: insights from a multicenter retrospective real‐world study

**DOI:** 10.1002/mco2.624

**Published:** 2024-06-19

**Authors:** Chunxiao Sun, Yijia Hua, Nan Jin, Xiaojia Wang, Jian Huang, Xinyu Wu, Tianyu Zeng, Xueqi Yan, Fan Yang, Yan Liang, Xiang Huang, Wei Li, Yongmei Yin

**Affiliations:** ^1^ Department of Oncology The First Affiliated Hospital of Nanjing Medical University Nanjing China; ^2^ Gusu School Suzhou Municipal Hospital, The Affiliated Suzhou Hospital of Nanjing Medical University Suzhou China; ^3^ The First Clinical College of Nanjing Medical University Nanjing China; ^4^ Department of Breast Medical Oncology Cancer Hospital of the University of Chinese Academy of Science Hangzhou China; ^5^ Department of Breast Surgery The Second Affiliated Hospital, Zhejiang University School of Medicine Hangzhou China; ^6^ Department of Oncology Sir Run Run Hospital of Nanjing Medical University Nanjing China; ^7^ Jiangsu Key Lab of Cancer Biomarkers, Prevention and Treatment, Collaborative Innovation Center for Cancer Medicine Nanjing Medical University Nanjing China

**Keywords:** HER2‐positive breast cancer, real‐world study, trastuzumab emtansine resistance, tyrosine kinase inhibitors

## Abstract

The use of trastuzumab emtansine (T‐DM1) has revealed significant efficacy in HER2‐positive metastatic breast cancer (MBC). However, optimal therapeutic strategies following T‐DM1 failure remain a subject of debate in clinical practice. In this multicenter, retrospective, real‐world study, we sought to examine the effectiveness and safety of tyrosine kinase inhibitors (TKIs) as a therapeutic strategy in HER2‐positive MBC who developed T‐DM1 resistance. Between September 2018 and December 2022, 66 patients were enrolled. The median progression‐free survival of TKIs‐based therapy was 10.1 months (95% CI, 4.7–15.6). Objective response rate and clinical benefit rate were 18.2 and 66.7%, respectively. TKIs‐based therapy demonstrated better effectiveness in patients who had previously derived benefit from T‐DM1 and featured acquired resistance to trastuzumab. The most common adverse events were diarrhea (36, 54.5%), hand‐foot syndrome (31, 47.0%), and leucopenia (30, 45.5%). In conclusion, TKIs‐based therapy showed promising effectiveness and safety in HER2‐positive MBC patients after T‐DM1 failure.

## Graphical abstract

TKIs‐based therapy could become an alternative treatment option for overcoming T‐DM1 resistance in HER2‐positive breast cancer.

Patients who benefited from previous T‐DM1 treatment ≥6 months and featured acquired resistance to trastuzumab might achieve a better survival in subsequent TKIs treatment.

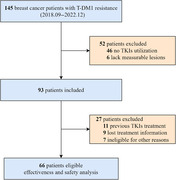

## 1 INTRODUCTION

Characterized by the overexpression of the human epidermal growth factor receptor 2 (HER2), HER2‐positive breast cancer accounts for approximately 20% of all breast cancer cases worldwide.^1^ The activation of HER2 triggers downstream signaling pathways and regulates various biological processes, which promote breast cancer cell proliferation, invasion, and metastasis.^2^ Amplification of HER2 is recognized as an indicator of hostile phenotype and related with unfavorable prognosis.^3^, ^4^ The combination of trastuzumab, pertuzumab, and taxane‐based chemotherapy has been recommended as the standard first‐line therapy for patients with HER2‐positive metastatic breast cancer (MBC).^5^, ^6^ T‐DM1, an antibody–drug conjugate (ADC) incorporating DM1, has been acknowledged as the preferred second‐line treatment of HER2‐positive MBC and postneoadjuvant therapy for HER2‐positive early breast cancer, demonstrating remarkable and durable clinical efficacy.^7^, ^8^, ^9^ However, disease progression still commonly happens under such a standard treatment.^10^ Although continuation of HER2‐targeted therapies is still preferred in clinical practice, no guidelines detailed recommendations for HER2‐postive MBC patients who experience disease progression after T‐DM1.

Presently, some studies have explored treatment strategies for heavily treated HER2‐postive MBC. Margetuximab, a chimeric antibody, provided a median progression‐free survival (PFS) of 5.8 months in patients with disease progression after two or more prior anti‐HER2 therapies.^11^ The NALA trial revealed that, compared with lapatinib, neratinib plus capecitabine improved PFS in HER2‐positive MBC patients who had received at least two HER2‐directed regimens, which therefore has been acknowledged as an effective third‐line treatment option for HER2‐positive MBC.^12^ The HER2CLIMB trial indicated that the dual HER2 blockade by combining tucatinib and trastuzumab significantly extended the PFS and overall survival (OS).^13^ Encouragingly, findings from the DESTINY‐Breast01 trial revealed a median PFS of 16.4 months with trastuzumab deruxtecan (T‐DXd) in HER2‐positive MBC patients previously treated with T‐DM1 and other anti‐HER2 targeted therapies.^14^ However, margetuximab, neratinib, and tucatinib have not been approved for MBC in China and T‐DXd is still unavailable to most Chinese patients on account of economic burden. Owing to factors including drug accessibility and other considerations, the commonly recommended anti‐HER2 regimens after T‐DM1 include various TKIs, such as lapatinib and pyrotinib.^15^

The EGF100151 study revealed that among HER2‐positive MBC patients who had experienced treatment failure with trastuzumab, lapatinib, and capecitabine could lead to improved PFS.^16^ Recent published studies indicated that the lapatinib‐based therapy might become an effective therapeutic option for HER2‐positive MBC previously treated with T‐DM1.^17^, ^18^ Pyrotinib, as a small molecular irreversible TKI inhibiting epidermal growth factor receptor (EGFR), HER2 and HER4, demonstrated significant improvements in PFS among patients with HER2‐positive MBC featuring trastuzumab and chemotherapy resistance.^19^, ^20^ The PHOEBE study showed that in patients with HER2‐positive MBC, pyrotinib and capecitabine achieved longer median PFS compared with lapatinib plus capecitabine.^19^ However, it is still controversial about the effectiveness and safety profile of TKIs‐based regimens in the context of T‐DM1 resistance.

Considering these circumstances, we embarked on a multicenter, real‐world study aimed at evaluating the effectiveness and tolerability of TKIs‐based therapy among individuals with HER2‐positive MBC who had developed resistance to T‐DM1. This investigation sought to shed light on the operation of TKIs in this specific cohort of patients, thereby contributing valuable insights to clinical practice and patient care.

## 2 RESULTS

### 2.1 Baseline characteristics

**FIGURE 1:**
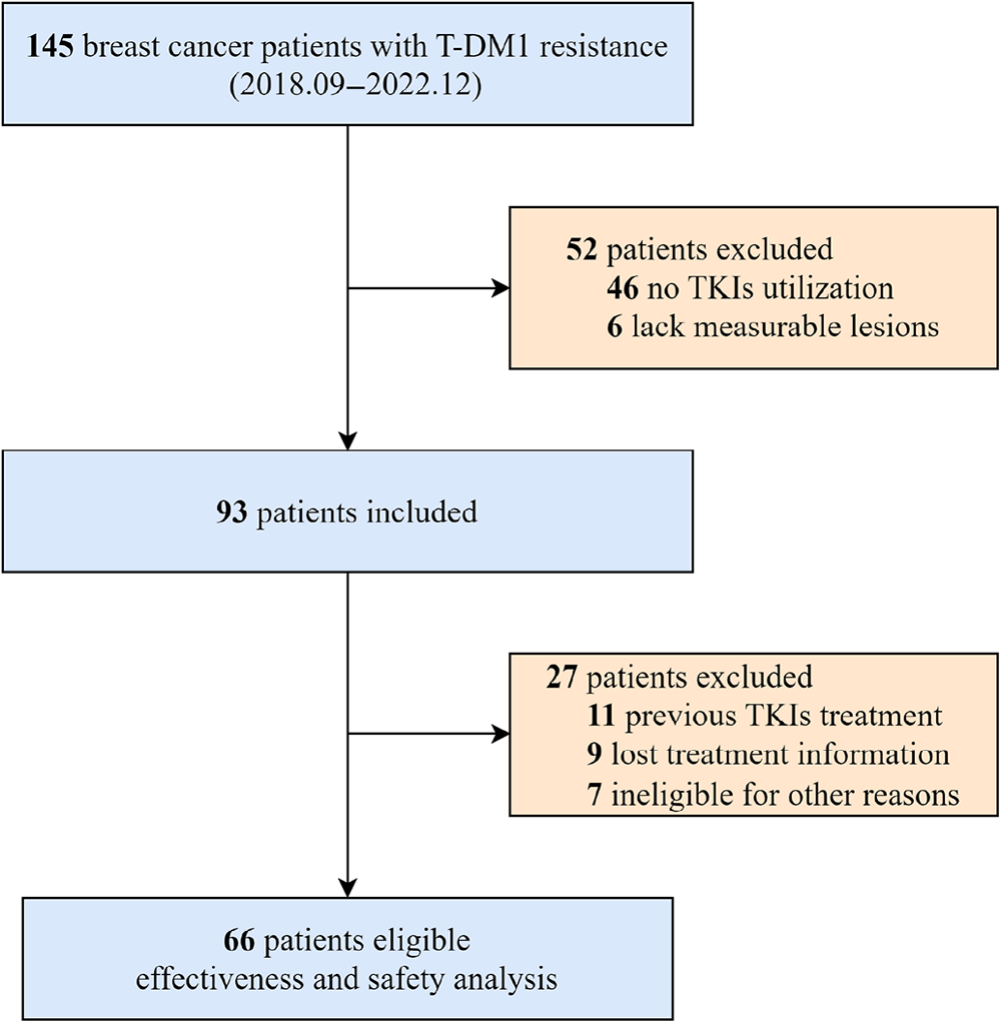
A flowchart detailing the process of patients’ selection. TKI, tyrosine kinase inhibitor.

Figure 1 illustrates the selection process for our real‐world study, which enrolled 66 patients diagnosed with HER2‐positive MBC. Table 1 provides a detailed overview of the comprehensive baseline clinical characteristics of the patients included in the study. Median age was 54 years (interquartile range, 49−59 years). A total of 27 (40.9%) patients were hormone receptor positive, while 39 (59.1%) patients were negative. In addition, 28 (42.4%) patients demonstrated primary resistance to trastuzumab, and 38 (57.6%) exhibited acquired resistance. Visceral metastases were detected in 44 patients (66.7%), while brain metastases were present in 15 (22.7%).

**TABLE 1:** Baseline characteristics of patients who received TKIs‐based therapy after T‐DM1 failure.

	TKI		
Characteristic	Lapatinib (*n* = 33)	Pyrotinib (*n* = 33)	N (%) (*n* = 66)
Age (years)			
Median (interquartile range)	54 (44–59)	55 (49–59)	54 (49–59)
Hormone receptor status			
Positive	12 (36.4)	15 (45.5)	27 (40.9)
Negative	21 (63.6)	18 (54.5)	39 (59.1)
Trastuzumab resistance			
Primary resistance	17 (51.5)	11 (33.3)	28 (42.4)
Acquired resistance	16 (48.5)	22 (66.7)	38 (57.6)
Visceral metastases			
Yes	23 (69.7)	21 (63.6)	44 (66.7)
No	10 (30.3)	12 (36.4)	22 (33.3)
Metastatic sites			
Brain	7 (21.2)	8 (24.2)	15 (22.7)
Lung	16 (48.5)	14 (42.4.)	30 (45.5)
Liver	8 (24.2)	10 (30.3)	18 (27.3)
Lymph nodes	21 (63.6)	22 (66.7)	43 (65.2)
Bone	7 (21.2)	9 (27.3)	16 (24.2)
Chest wall	6 (18.2)	3 (9.1)	9 (13.6)
PFS of T‐DM1 (months)			
<6.0	20 (60.6)	14 (42.4)	34 (51.5)
≥6.0	13 (39.4)	17 (51.5)	30 (45.5)
Unknown	0 (0.0)	2 (6.1)	2 (3.0)
Lines of TKIs‐based therapy			
2	8 (24.2)	7 (21.2)	15 (22.7)
≥3	25 (75.8)	26 (78.8)	51 (77.3)
Therapy regimens			
TKIs	1 (3.0)	0 (0.0)	1 (1.5)
TKIs + capecitabine	25 (75.8)	21 (63.6)	46 (69.7)
TKIs + trastuzumab + capecitabine	4 (12.1)	2 (6.1)	6 (9.1)
TKIs + trastuzumab + vinorelbine	2 (6.1)	0 (0.0)	2 (3.0)
Others	1 (3.0)	10 (30.3)	11 (16.7)

Abbreviations: TKI, tyrosine kinase inhibitor; T‐DM1, trastuzumab emtansine.

T‐DM1 treatment was administered to 34 patients (51.5%) less than 6 months prior, while 30 patients (45.5%) had received T‐DM1 treatment 6 months or more. In total, 15 (22.7%) patients received second‐line treatments based on TKIs, while 51 (77.3%) received third‐line or later therapies. A total of 54 (81.8%) patients received TKIs in combination with capecitabine, vinorelbine, or trastuzumab, while 1 (1.5%) patient received TKIs alone.

### 2.2 Effectiveness

**FIGURE 2:**
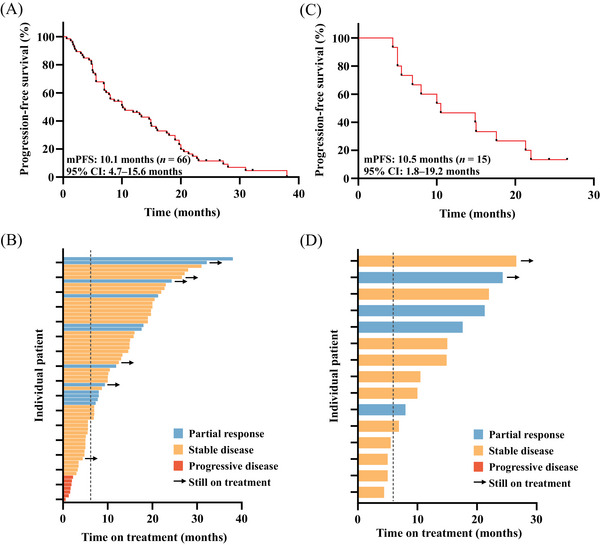
The treatment response and Kaplan–Meier analysis of patients who received TKIs‐based therapies following T‐DM1 failure. (A) PFS analysis of all patients treated with TKIs‐based therapies. (B) Treatment response of TKIs‐based therapies in all patients. The dashed line denotes 6 months thresholds for clinical benefits. (C) PFS analysis of patients with brain metastases treated with TKIs‐based therapies. (D) Treatment response of TKIs‐based therapies in patients with brain metastases. The dashed line denotes 6 months thresholds for clinical benefits. PFS, progression‐free survival; T‐DM1, trastuzumab emtansine; TKI, tyrosine kinase inhibitor.

At the cutoff date (December 31, 2023), the median follow‐up was 32.2 months (range, 24.0–40.3). Disease progression was observed in 60 patients (90.9%), while 15 patients (22.7%) had succumbed to death. The median PFS was 10.1 months (95% confidence interval [CI] 4.7–15.6) in all patients (Figure 2A). OS data were not mature. Objective response rate (ORR) and clinical benefit rate (CBR) were 18.2 and 66.7%, respectively. A total of 12 (18.2%) patients received partial response (PR), 47 (71.2%) patients received stable disease (SD) and no patients received complete response (CR) (Figure 2B). For patients with brain metastasis (*n* = 15), the median PFS was 10.5 months (95% CI 1.8–19.2) (Figure 2C). Intracranial ORR was 26.7% and CBR reached 73.3%. Four patients (26.7%) received PR, 11 patients (73.3%) received SD, and no patients received CR (Figure 2D).

**FIGURE 3:**
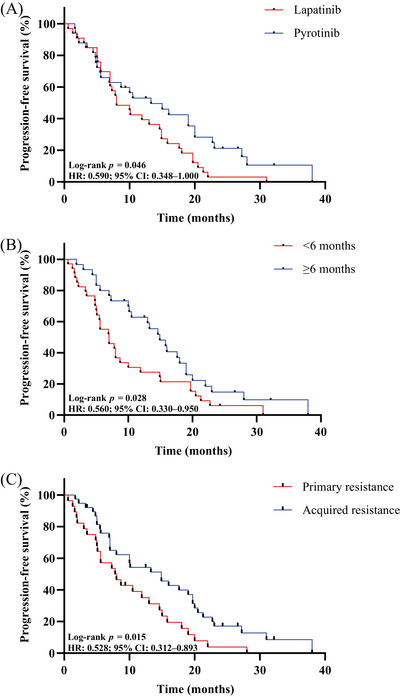
Kaplan–Meier analysis of TKIs in patients with different clinical characteristics. (A) PFS of patients who received lapatinib and pyrotinib. (B) PFS of patients who benefited from T‐DM1 < 6 months and ≥6 months. (C) PFS of patients with different response to trastuzumab. TKI, tyrosine kinase inhibitor; T‐DM1, trastuzumab emtansine.

**FIGURE 4:**
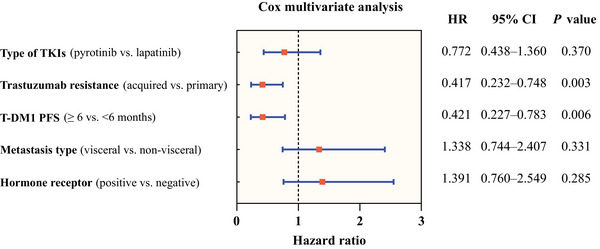
Cox multivariate analysis of factors linked with PFS in TKIs‐based therapies. TKI, tyrosine kinase inhibitor; T‐DM1, trastuzumab emtansine; PFS, progression‐free survival; HR, hazard ratio.

The log‐rank test results revealed that age, hormone receptor status, metastasis type, brain metastasis, lung metastasis, liver metastasis, bone metastasis, lymph nodes metastasis, and lines of TKIs‐based therapy were not associated to the effectiveness of TKIs (Table 2). Pyrotinib (*n* = 33) demonstrated superior PFS compared with lapatinib (*n* = 33) (13.3 months vs. 8.0 months, *p *= 0.046) (Figure 3A). Patients who experienced a benefit from T‐DM1 treatment for at least 6 months demonstrated a longer PFS after TKIs‐based therapy (14.9 months vs. 7.0 months, *p *= 0.028) (Figure 3B). Meanwhile, TKIs‐based therapy yielded a better PFS in patients featuring acquired resistance to trastuzumab (14.9 months vs. 7.8 months, *p *= 0.015) (Figure 3C). The multivariate analysis (Figure 4) corroborated that previous T‐DM1 PFS ≥6 months could be a predictor for better PFS (*p *= 0.006, hazard ratio [HR] 0.421, 95% CI 0.227−0.783), so as acquired resistance to trastuzumab (*p *= 0.003, HR 0.417, 95% CI 0.232−0.748). The sensitivity analyses yielded results that were largely consistent with the findings mentioned above. (Figure S1 and Table S1)

**TABLE 2:** Log‐rank analysis of factors associated with TKIs PFS.

Characteristic	Log‐rank analysis
	*p* Value
Age (≥50 vs. < 50)	0.767
Type of TKIs (pyrotinib vs. lapatinib)	0.046
Hormone receptor (positive vs. negative)	0.747
Trastuzumab resistance (acquired vs. primary)	0.015
Metastasis type (visceral vs. nonvisceral)	0.589
Brain metastasis (yes vs. no)	0.697
Lung metastasis (yes vs. no)	0.374
Liver metastasis (yes vs. no)	0.347
Bone metastasis (yes vs. no)	0.626
Lymph nodes metastasis (yes vs. no)	0.714
Lines of TKIs‐based therapy (≥3 vs. 2)	0.742
T‐DM1 PFS (≥6 vs. < 6 months)	0.028

Abbreviations: PFS, progression‐free survival; T‐DM1, trastuzumab emtansine; TKI, tyrosine kinase inhibitor.

### 2.3 Safety

The most common adverse events (AE) were diarrhea (36, 54.5%), hand‐foot syndrome (31, 47.0%), leucopenia (30, 45.5%), neutrocytopenia (21, 31.8%), anemia (20, 30.3%), and thrombocytopenia (15, 22.7%). The most common AEs of grade 3 or 4 were diarrhea (four, 6.1%), hand‐foot syndrome (three, 4.5%), leucopenia (two, 3.0%), and neutrocytopenia (two, 3.0%). Compared with lapatinib, pyrotinib showed similar tolerance totally, of which diarrhea (16 [48.5%] in lapatinib vs. 20 [60.6%] in pyrotinib) and hand‐foot syndrome (14 [42.4%] in lapatinib vs. 17 [51.5%] in pyrotinib) were the most common. In the lapatinib group, five out of 33 patients (15.2%) experienced AEs of grade 3 or worse, while in the pyrotinib group, this was observed in eight out of 33 patients (24.2%). There were no treatment‐related deaths. Table 3 provides an overview of the safety profile associated with therapy utilizing TKIs.

**TABLE 3:** Adverse events of patients who received TKIs‐based therapy after T‐DM1 failure.

	Lapatinib *n* (%)		Pyrotinib *n* (%)		Summary *n* (%)		
Adverse events	Grade 1−2	Grade 3−4	Grade 1−2	Grade 3−4	Grade 1−2	Grade 3−4	Any Grade
Diarrhea	15 (45.5)	1 (3.0)	17 (51.5)	3 (9.1)	32 (48.5)	4 (6.1)	36 (54.5)
Hand‐foot syndrome	13 (39.4)	1 (3.0)	15 (45.5)	2 (6.1)	28 (42.4)	3 (4.5)	31 (47.0)
Leucopenia	16 (48.5)	2 (6.1)	12 (36.4)	0 (0.0)	28 (42.4)	2 (3.0)	30 (45.5)
Neutrocytopenia	9 (27.3)	1 (3.0)	10 (30.3)	1 (3.0)	19 (28.8)	2 (3.0)	21 (31.8)
Anemia	9 (27.3)	0 (0.0)	11 (33.3)	0 (0.0)	20 (30.3)	0 (0.0)	20 (30.3)
Thrombocytopenia	9 (27.3)	0 (0.0)	5 (15.2)	1 (3.0)	14 (21.2)	1 (1.5)	15 (22.7)
Increased alanine or aspartate aminotransferase	5 (15.2)	0 (0.0)	2 (6.1)	0 (0.0)	7 (10.6)	0 (0.0)	7 (10.6)
Nausea	3 (9.1)	0 (0.0)	3 (9.1)	0 (0.0)	6 (9.1)	0 (0.0)	6 (9.1)
Hyperbilirubinemia	3 (9.1)	0 (0.0)	1 (3.0)	0 (0.0)	4 (6.1)	0 (0.0)	4 (6.1)
Rash	1 (3.0)	0 (0.0)	1 (3.0)	1 (3.0)	2 (3.0)	1 (1.5)	3 (4.5)
Hyperlipoidemia	1 (3.0)	0 (0.0)	1 (3.0)	0 (0.0)	2 (3.0)	0 (0.0)	2 (3.0)
Fever	1 (3.0)	0 (0.0)	0 (0.0)	0 (0.0)	1 (1.5)	0 (0.0)	1 (1.5)
Hemafecia	1 (3.0)	0 (0.0)	0 (0.0)	0 (0.0)	1 (1.5)	0 (0.0)	1 (1.5)
Alopecia	0 (0.0)	0 (0.0)	1 (3.0)	0 (0.0)	1 (1.5)	0 (0.0)	1 (1.5)

Abbreviations: TKI, tyrosine kinase inhibitor; T‐DM1, trastuzumab emtansine.

## 3 DISCUSSION

Over past decades, significant improvements have been achieved in HER2‐positive MBC treatment,^21^ but outcomes of heavily pretreated patients are still undesirable. Due to drug approval, tucatinib and neratinib were not available for HER2‐positive MBC in China, resulting the lack of evidential regimens for patients after T‐DM1 failure. To provide more reliable evidence in clinical practice, we conducted this multicenter, retrospective, real‐world study to assess the effectiveness of TKIs‐based treatment in Chinese patients previously treated with T‐DM1.

Our research observed the practice of sequential treatment of TKIs‐based therapy after T‐DM1 progression was effective, patients who have brain metastases also benefited from this regimen. The duration of prior T‐DM1 treatment and types of trastuzumab resistance were significantly associated with the effectiveness of TKIs‐based therapy, yet no relationship was observed between TKIs effectiveness and hormone receptor status, metastatic sites, metastatic type, or number of TKIs lines.

In the EGF100151 study, lapatinib plus capecitabine can bring about a median PFS of 8.4 months.^16^ In the PHENIX and PHOEBE study, the median PFS of pyrotinib with capecitabine was reported as 11.1 and 12.5 months, respectively.^19^, ^20^ The majority of patients in the aforementioned studies had received two or fewer treatment lines, and none had been previously treated with T‐DM1. It is crucial to note that 77.3% patients received third‐line or later treatments based on TKIs in our study, which indicated that there was a greater tendency for TKIs to be used in patients with heavy pretreatment. In a retrospective, multicenter real‐world study involving 168 patients diagnosed with HER2‐positive MBC, treatment with pyrotinib‐based therapy demonstrated a median PFS of 8.00 months and a median OS of 19.07 months.^22^ In another previous retrospective study of patients with heavily pretreated HER2‐positive MBC, trastuzumab and lapatinib combined chemotherapy achieved a promising PFS of 10.9 months.^23^ Meanwhile the median PFS with pyrotinib and lapatinib treatment in our study was 13.3 and 8.0 months, respectively, which was numerically comparable with the median PFS results of pyrotinib and lapatinib in above studies. Even though the results of this study may have been biased by selection bias, they provide evidence to supplement other clinical trials that have not yet been conducted and prompt that TKIs can engender an encouraging therapeutic effect in HER2‐positive MBC previously treated with T‐DM1.

The evolution of anti‐HER2 therapies has broadened the therapeutic landscape for breast cancer patients with brain metastases. Different from monoclonal antibodies, TKIs seem more inclined to penetrate the blood–brain barrier owing to their physical characteristics, such as smaller molecular weight.^24^ The median PFS for patients with brain metastases who received lapatinib and capecitabine was 5.5 months.^25^ In the PERMEATE study, pyrotinib and capecitabine demonstrated a median PFS of 11.3 months in patients with radiotherapy‐naive HER2‐positive brain metastases.^26^ Another real‐world study also confirmed the effectiveness of pyrotinib‐based in HER2‐positive MBC patients with brain metastases.^27^ Meanwhile, our study reported a 10.5‐months PFS for patients with brain metastasis, which was approximate with that reported in above studies. Although the sample size is limited, these data suggest that TKIs might become a competent choice for brain metastases after T‐DM1 failure.

The influence of hormone receptor status on the efficacy of TKIs is discordant. In the PHENIX study, negative hormone receptor status was associated with improved PFS and enhanced response to pyrotinib plus capecitabine.^20^ Similarly, the PFS benefit from neratinib plus capecitabine was observed in the hormone receptor‐negative subgroup rather than the positive subgroup.^28^ However, improved invasive disease‐free survival of neratinib in ExteNET trial was observed in hormone receptor‐positive group.^29^ In our investigation, no significant difference of PFS was indicated in the comparison between hormone receptor‐positive and ‐negative subgroup. This result was consistent with the real‐world data of PRETTY trial, which indicated pyrotinib‐based regimens could result in similar PFS in different hormone receptor status.^30^ Considering the interaction between estrogen receptor and the human epidermal growth factor receptor family,^31^ dual hormone receptor and HER2 pathway targeted therapies could be a rational regimen of maintenance therapy for hormone receptor‐positive/HER2‐positive MBC.

Some studies assessed the benefit of TKIs‐based therapy in view of the response to previous treatment such as trastuzumab. The PHENIX study indicated that pyrotinib and capecitabine could benefit both trastuzumab primary and acquired resistant patients.^20^ Some real‐world data indicated that pyrotinib improved survival outcomes irrespective of trastuzumab response in HER2‐positive MBC.^32^ In our study, patients with acquired resistance to trastuzumab achieved significantly better PFS compared with patients with primary resistance to trastuzumab. Significantly, our results indicated that patients who benefited from T‐DM1 more than 6 months previously experienced an extended survival in the subsequent TKIs‐based therapy, which imply that the effectiveness of TKIs‐based therapy might be associated with the response to T‐DM1 treatment.

Our study demonstrated that TKIs‐based therapy tended to be well tolerated by patients with T‐DM1 resistance. The common AEs include diarrhea (54.5%), hand‐foot syndrome (47.0%), and leucopenia (45.5%), which were consistent with previous retrospective studies of TKIs in HER2‐positive MBC.^23^, ^33^ Moreover, the AEs of ≥3 grade were documented in approximately 6.1% of patients, including diarrhea (6.1%), hand‐foot syndrome (3, 4.5%), and leucopenia (1, 3.0%). Significantly, the incidence of thrombocytopenia (22.7%) was higher than that reported in previous prospective and retrospective studies.^12^, ^23^, ^33^, ^34^ The increased incidence of thrombocytopenia might be attributed to the administration of T‐DM1, as previous reports have indicated that T‐DM1‐induced thrombocytopenia is largely a result of DM1‐induced impairment of megakaryocyte differentiation.^35^ Thereby, the potential toxicity of the sequential use of TKIs after T‐DM1 must be considered in clinical practice and a comprehensive evaluation of the safety of the subsequent TKIs‐based therapy following ADCs is required in the future.

Our study is the first to investigate the real‐world effectiveness of TKIs‐based treatment in patients previously exposed to T‐DM1. Clinical trials of anti‐HER2 TKIs among Chinese population accrued patients who received trastuzumab and pertuzumab, but none who received prior T‐DM1, so our study would offer pivotal supplement to current clinical trials. Furthermore, our results reported the promising effectiveness of TKIs in T‐DM1 failure, which holds significant importance for the management of patients and furnishes a theoretical foundation for physicians.

Despite the study's strengths, it also has some limitations. First, our study was a retrospective evaluation of patients in the real‐world practice, thus the results of our study were limited in comparison with randomized controlled trials. The retrospective design might not offer the same level of methodological robustness as randomized, so the selection bias was present, and the recall bias was inevitable. A prospective cohort study is necessary to further validate our conclusion and efforts are underway. In addition, due to patient adherence, financial limitations, and considerations regarding quality of life, the sample size was quite small. Further, several exposure‐derived endpoints that can be derived on structured data were missed in our study due to the lack of data. Moreover, more standardized study endpoints for recurrent disease to assess clinical benefit are critical such as growth modulation index. Finally, the duration of follow up was relatively short and the median OS has not yet reached, but follow‐up is ongoing.

In conclusion, TKIs‐based therapy was well tolerated and demonstrated promising effectiveness in patients with TDM1‐resistant HER2‐positive MBC. Using TKIs after T‐DM1 treatment could also benefit patients with brain metastases. These findings provide support for ongoing clinical trials and warrant further evaluation to confirm TKIs‐based therapy's effectiveness in patients with T‐DM1 resistance.

## 4 METHODS

### 4.1 Study design and patient eligibility

This study is a multicenter, retrospective, real‐world study (register number: NCT05231863), which is conducted in The First Affiliated Hospital of Nanjing Medical University and Cancer Hospital of The University of Chinese Academy of Sciences. The study received approval from the Ethics Committee and Institutional review board of the First Affiliated Hospital of Nanjing Medical University and conducted in consistent with the guidelines of Good Clinical Practice and the Declaration of Helsinki. The data collection was conducted by physicians and followed the ethical standards of each of the participating institutions.

From September 2018 to December 2022, 66 patients with HER2‐positive MBC were enrolled, and the last follow‐up was December 31, 2023. The eligibility criteria included: (i) females aged 18−70 years; (ii) confirmed pathologic diagnosis of HER2‐positive MBC (immunochemistry 3+, or 1/2+ with positive results of fluorescence in situ hybridization); (iii) presence of at least one measurable metastatic lesion as defined by the Response Evaluation Criteria in Solid Tumors guidelines version 1.1 (RECIST 1.1); (iv) initiation of TKIs‐based regimens following disease progression of T‐DM1 in advanced setting. Patients were excluded if they had been previously treated with TKIs, or lost treatment information. Figure 1 illustrates the patient selection process in detail, outlining the criteria and steps involved. Trastuzumab primary resistance was characterized as disease recurrence occurring during or after (less than 12 months) adjuvant trastuzumab or disease progression identified within 3 months after first‐line trastuzumab. The definition of acquired resistance to trastuzumab involves disease recurrence that is discovered after (more than 12 months) completion of adjuvant trastuzumab, or disease progression after two or more lines of trastuzumab, which responded or stabilized during the initial treatment.^36^ The line of therapy was identified as a serial chronological number of each administered systemic anti‐cancer therapy in advanced refractory cancer treatment settings, which consisted of one or more anti‐cancer agents used alone or in combination or sequence in repeating cycles.^37^

### 4.2 Treatments

All patients received T‐DM1 treatment in the stage of metastasis and/or recurrence. After disease progression, patients received TKIs‐based therapies, including lapatinib or pyrotinib. Initial dose, dose adjustment, discontinuation, combined chemotherapy, and/or combined HER2‐targeted therapy were administrated by physicians and physician–patient communication. The length of one cycle for TKIs treatment was 21 days.

### 4.3 Outcomes

PFS served as the primary endpoint, denoting the duration from the commencement of TKIs treatment to either disease progression or death from any cause. ORR, CBR, OS, and safety constituted the secondary endpoints. ORR was determined by assessing the percentage of patients who attained an objective response (CR or PR); CBR was determined by evaluating the proportion of patients under disease control (CR, PR, or SD for a minimum of 24 weeks); OS was defined as the time from drug administration to death by any cause. The treatment response was assessed according to the RECIST 1.1 system. According to the Common Terminology Criteria for Adverse Events version 5.0, AEs were graded.

### 4.4 Statistical analysis

Descriptive statistics were used to analyze clinicopathologic characteristics. Numerical data were depicted using median, mean, and interquartile range, while categorical data were delineated by proportions and rates. PFS and OS of all patients, patients with brain metastases, patients receiving lapatinib or pyrotinib, and patients with different T‐DM1 responses were calculated using Kaplan–Meier methods. Survival curves in patients grouped by variables of interest, such as TKIs kinds and time of benefit derived from T‐DM1 ≥6 or < 6 months, were compared by the log‐rank test. To minimize bias, potential confounders were identified and variables to be included in the models were specified using directed acyclic graphs (Figure S2). Factors associated with all effectiveness outcomes which defined as log‐rank test *p* < 0.2 were included in multivariate analyses. The HR with 95%CI was analyzed using the Cox proportional hazard model. The significance of all tests was defined as *p* < 0.05, with two‐sided tests performed on all data. SPSS 26.0, and GraphPad Prism 9.0, was used for statistical evaluations.

## AUTHOR CONTRIBUTIONS

*Conceptualization*: Chunxiao Sun, Wei Li, and Yongmei Yin. *Investigation*: Xiaojia Wang, Jian Huang, Fan Yang, Yan Liang, and Xiang Huang. *Methodology*: Chunxiao Sun, Xinyu Wu, Tianyu Zeng, and Xueqi Yan. *Resources*: Xiaojia Wang, Jian Huang, and Xinyu Wu. *Data curation, formal analysis, and writing*—original draft: Yijia Hua and Nan Jin. *Writing—review and editing*: Chunxiao Sun, Wei Li, and Yongmei Yin. *Supervision and project administration*: Wei Li and Yongmei Yin. All authors have read and approved the final manuscript.

## CONFLICT OF INTEREST STATEMENT

The authors declare that there is no conflict of interest.

## ETHICS STATEMENT

This study was approved by the Ethics Committee of the First Affiliated Hospital of Nanjing Medical University (Approved No: 2018‐SRFA‐154). This study was registered at clinicaltrials.gov (NCT05231863).

## Supporting information

Supporting Information

## Data Availability

All data analyzed in the study are included in the article. Further inquiries can be directed to the corresponding authors.
