# Supplementary material for: Tyrosine kinase inhibitors in HER2‐positive metastatic breast cancer with trastuzumab emtansine resistance: insights from a multicenter retrospective real‐world study
Source: MedComm (2020). 2024 Jun 19;5(7):e624. doi: 10.1002/mco2.624 (PMC11187841; doi:10.1002/mco2.624)
Supplement: Supplementary file 1 — Supporting Information [file MCO2-5-e624-s001.docx]

**Supplementary Information**

Tyrosine Kinase Inhibitors in HER2-Positive Metastatic Breast Cancer with Trastuzumab Emtansine Resistance: Insights from a Multicenter Retrospective Real-World Study

Chunxiao Sun, Yijia Hua, Nan Jin, Xiaojia Wang, Jian Huang, Xinyu Wu, Tianyu Zeng, Xueqi Yan, Fan Yang, Yan Liang, Xiang Huang, Wei Li, Yongmei Yin


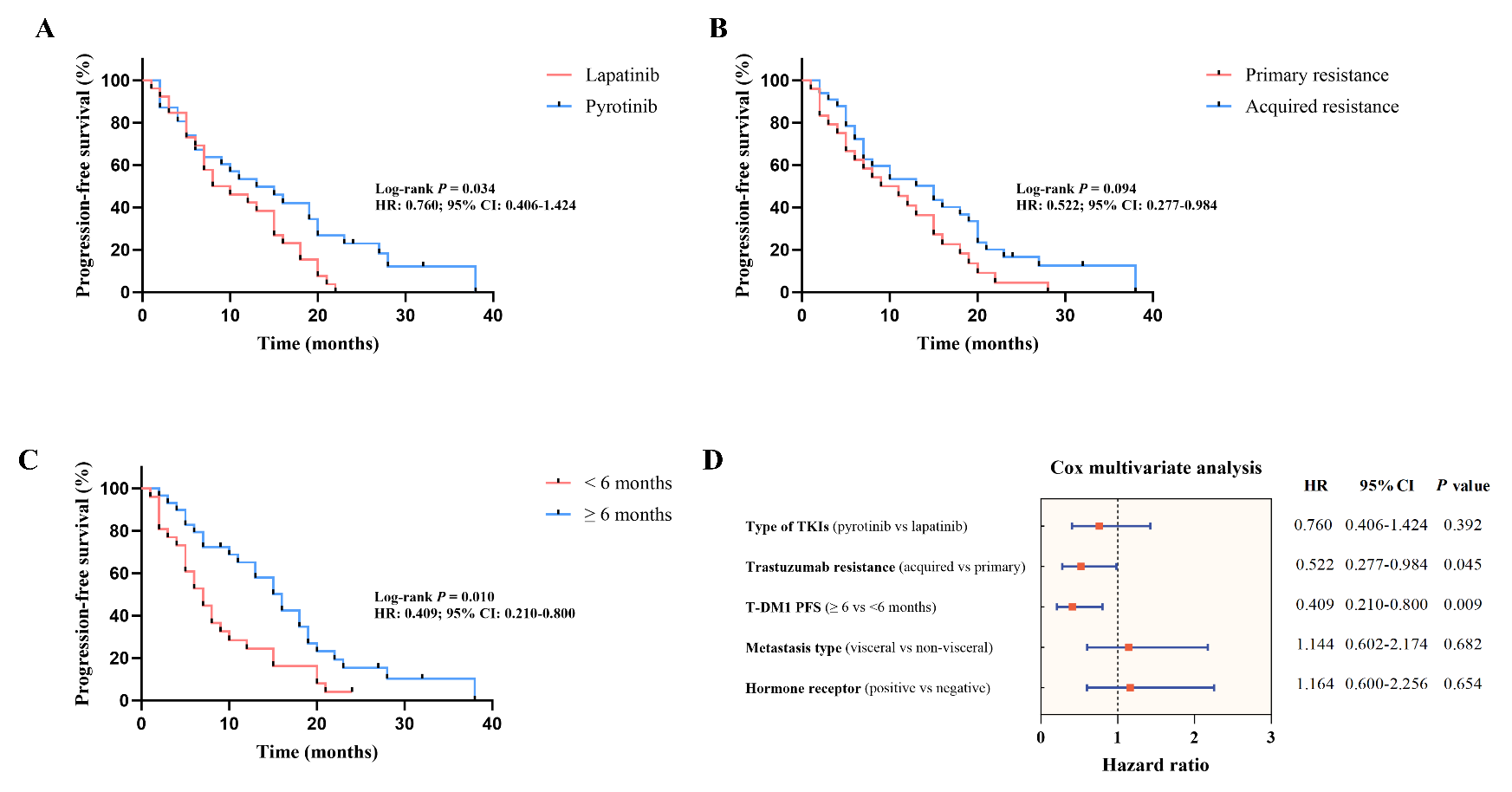


**Supplementary Figure S1.** Sensitivity analyses of the effectiveness of TKIs plus chemotherapy in HER2-positive MBC with T-DM1 resistance. (A) PFS of patients receiving treatment with lapatinib and pyrotinib. (B) PFS of patients with primary and acquired resistance to trastuzumab. (C) PFS of patients receiving clinical benefit from T-DM1＜6 months and ≥6 months. (D) Cox multivariate analysis of factors associated with PFS in TKIs plus chemotherapy. TKI: tyrosine kinase inhibitor; T-DM1: trastuzumab emtansine; PFS: progression-free survival; HR: hazard ratio.

**
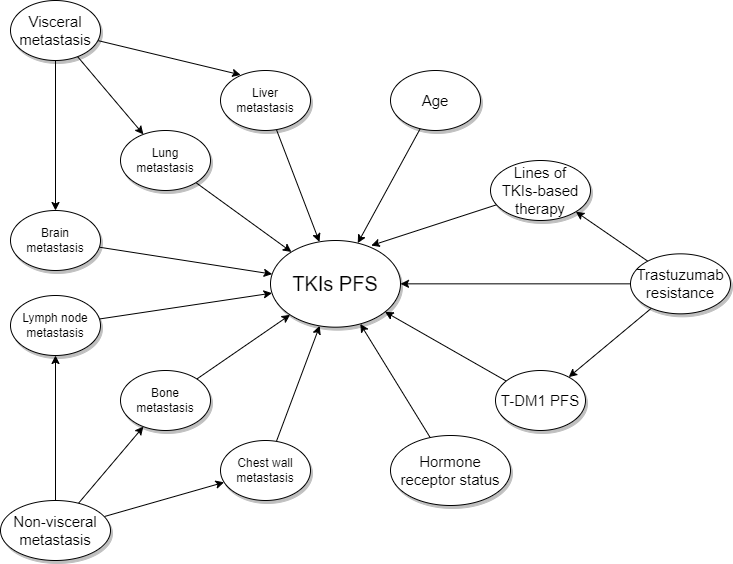
**

**Supplementary Figure S2.** Directed acyclic graphs which showed confounders associated with TKIs PFS. TKI: tyrosine kinase inhibitor; T-DM1: trastuzumab emtansine; PFS: progression-free survival.

| **Characteristic** | **Log-rank analysis** |
| --- | --- |
|  | ***P* value** |
| Age (≥50 vs <50) | 0.445 |
| Type of TKIs (pyrotinib vs lapatinib) | 0.034 |
| Hormone receptor (positive vs negative) | 0.446 |
| Trastuzumab resistance (acquired vs primary) | 0.094 |
| Metastasis type (visceral vs non-visceral) | 0.817 |
| Brain metastasis (yes vs no) | 0.580 |
| Lung metastasis (yes vs no) | 0.321 |
| Liver metastasis (yes vs no) | 0.838 |
| Bone metastasis (yes vs no) | 0.820 |
| Lymph nodes metastasis (yes vs no) | 0.498 |
| Lines of TKIs-based therapy (≥3 vs 2) | 0.700 |
| T-DM1 PFS (≥6 vs <6 months) | 0.010 |

**Supplementary Table S1**. Log-rank test in the sensitivity analysis regarding factors associated with PFS in TKIs plus chemotherapy.

*TKI: tyrosine kinase inhibitor; T-DM1: trastuzumab emtansine; PFS: progression-free survival.
